# The meaning of a word

**DOI:** 10.7554/eLife.97360

**Published:** 2024-02-29

**Authors:** Kirsty Lauder

**Affiliations:** 1 Cornell Lab of Ornithology, Cornell University Ithaca United States

**Keywords:** sparks of change, research culture, neurodiversity, being neurodivergent in academia, neuroinclusivity

## Abstract

A fraught exchange on social media leads a PhD student to reconsider how she conducts research on neuroinclusivity while being neurodivergent herself.

I read her WhatsApp message as I head to the Victoria line, my favourite on the London Underground: “Maybe add a sentence or two to explain what neurodiversity is?” I look again at the tweet my friend is referring to and realise she is probably right. I’m trying to find participants for my e-learning programme on neurodiversity for managers and human resources professionals; I’ve spent months of my PhD designing this study, but I’ve only shared the recruitment poster for it. I quickly add two sentences to my post before I hop on the train and lose signal. I won’t have time to check Twitter for the rest of the day. In fact, I’m planning to work late as I feel I need to compensate for my appointment yesterday, the appointment I had waited two years for and which had confirmed what I’ve known for a while: I am neurodivergent myself.

I was finishing my master’s degree in occupational psychology when that thought first entered my mind. A fresh psychology graduate, I had started my first ‘proper’ job supporting students with special educational needs for the local council. As I was writing plans detailing the adjustments these children needed at school, I couldn’t help noticing how similar I was to some of them; I also began questioning what would happen once they had left educational settings. Eager to know more, I embarked on a PhD exploring the effectiveness of workplace accommodations for ADHDers – and my own diagnostic journey.

I settle at my desk, ready to spend my day writing, teaching and dutifully trying to ignore how apprehensive I am about finding enough volunteers for my programme. I’m data hungry – the more participants, the better the findings. I’ve posted about it with all the hashtags on every social media platform I can think of. This is my last study. After three exhausting years that have fuelled my self-doubt and anxiety, I’m flirting with the idea I may finish this PhD after all.

I’m packing my things to head home when I finally open Twitter again. Amongst the retweets from friends and colleagues, some comments startle me. Neurodivergent academics and students are concerned about the way I defined ‘neurodiversity’ as an umbrella term for different types of neurodivergence, and how I used ‘neurodiverse’ instead of ‘neurodivergent’ to refer to individuals. They point to the fact that neurodiversity includes everyone, neurodivergent and neurotypical people alike, and that my definition does not reflect this.

I sit back down and continue scrolling. People are wondering how I could have created a good programme about neurodiversity if I couldn’t even explain the term correctly. They are questioning whether I have engaged with the neurodivergent community to build it, but also my expertise, my intelligence and ultimately whether my research should be trusted. My head starts to spin as a wave of panic and hurt washes over me. Many people are vouching for me online, but all I can see are the criticisms.

Angrily, irrationally, I begin to reply. I try to explain my research and my approach without giving away too much of the study, so I don’t introduce bias. The responses get more and more hurtful and I’m not thinking through mine. I’m so numb that I don’t even notice I’m on the underground again, heading home. All I can think about is that their words may be true, that my research is rubbish, that I am stupid, a failure, and causing harm to the very community for which I was trying to make the world a better place; the community I had looked forward to belonging to now that I had my formal diagnosis. Sitting on a bench in the middle of Victoria station, I call my parents and cry. The emotional impact of the rejection and criticism followed me through the remainder of my PhD.

It took me a while to acknowledge that I had indeed defined neurodiversity wrong. In hindsight, I think that everything felt very personal at the time because my own diagnosis was so fresh and emotional. My appointment had felt like undressing my mind in front of a stranger, the end of a journey marred by ableist remarks from healthcare professionals. Every detail of my childhood, friendships and mental health had been combed through, leaving me vulnerable, exhausted and questioning everything about myself. I was so focused on the personal aspect of the replies that I couldn’t hear their actual message: the importance of language and of engaging with the community.

I was aware of these topics when I posted about my study, but I hadn’t fully realised what was at stake. Slowly, the feedback I received encouraged me to delve deeper into the history of language and disability. I learned how the concept of neurodiversity emerged from the disability and autism rights movements, which aimed to emphasize the need to embrace the diverse ways, both neurodivergent and neurotypical, by which people experience and interact with the world. I could finally fully appreciate why using these terms incorrectly could generate frustration and concerns from a community whose voices and lived experiences have historically been considered as secondary to the opinions of academic or medical ‘experts’.

I discovered a different vocabulary related to neurodivergence and started to participate in fruitful debates about who owns the right to language, and who gets to decide which term is ‘right’. As I broadened the materials I used for work, I began to realise how much of the theories, frameworks and literature I had been taught as a psychology student had been shaped by the idea that differences are automatically pathological defects. I thought I knew about neurodiversity; I couldn’t have been more wrong.

Now is an exciting time for the neurodivergent community, with the nature of neurodiversity as a paradigm and concept constantly evolving. By encouraging a shift from focusing on ‘curing’ individuals towards supporting them instead, the movement opens the possibility to create real changes in schools and workplaces. As a neurodivergent person who simply wants to be my best self in environments traditionally designed for the neurotypical, this gives me hope. As an academic, it led me to my current postdoctoral project on neuroinclusivity, where I investigate how inclusion of neurodivergent and disabled students or staff can be improved in a conservation biology lab in the United States.

More importantly, these conversations encouraged me to change how I work. I learned to take a step back and seek feedback from others – no more tweets written rushing between trains! I actively talk about language, and I engage with academics outside my field to keep up to date with the neurodiversity vocabulary. I also re-examined what I thought I knew about conducting participatory research. Involving the community means that scientific questions and ideas start with them; it influences so many aspects of the research process, from how you establish working agreements or run meetings to how you communicate and produce materials that people can actually use. Now, an advisory group guides my work and its outputs. Their research contributions have been invaluable, but they have also supported me in ways that they may not know. When I face challenges and get frustrated with my progress, they are the ones who give me the motivation to keep going.

## About this article

This Sparks of Change article is part of a series of articles on being neurodivergent in academia, which includes a list of tips, resources and tools collated by neurodivergent scientists.

